# The Effectiveness of RNAi in *Caenorhabditis elegans* Is Maintained during Spaceflight

**DOI:** 10.1371/journal.pone.0020459

**Published:** 2011-06-01

**Authors:** Timothy Etheridge, Kanako Nemoto, Toko Hashizume, Chihiro Mori, Tomoko Sugimoto, Hiromi Suzuki, Keiji Fukui, Takashi Yamazaki, Akira Higashibata, Nathaniel J. Szewczyk, Atsushi Higashitani

**Affiliations:** 1 Division of Clinical Physiology, Royal Derby Hospital, University of Nottingham, Derby, England; 2 Graduate School of Life Sciences, Tohoku University, Sendai, Japan; 3 Advanced Engineering Services Co., Ltd., Tsukuba, Japan; 4 ISS Science Project Office, Institute of Space and Astronautical Science, Japan Aerospace Exploration Agency, Tsukuba, Japan; 5 Japan Space Forum, Ohtemachi, Chiyoda-ku, Japan; Brown University, United States of America

## Abstract

**Background:**

Overcoming spaceflight-induced (patho)physiologic adaptations is a major challenge preventing long-term deep space exploration. RNA interference (RNAi) has emerged as a promising therapeutic for combating diseases on Earth; however the efficacy of RNAi in space is currently unknown.

**Methods:**

*Caenorhabditis elegans* were prepared in liquid media on Earth using standard techniques and treated acutely with RNAi or a vector control upon arrival in Low Earth Orbit. After culturing during 4 and 8 d spaceflight, experiments were stopped by freezing at −80°C until analysis by mRNA and microRNA array chips, microscopy and Western blot on return to Earth. Ground controls (GC) on Earth were simultaneously grown under identical conditions.

**Results:**

After 8 d spaceflight, mRNA expression levels of components of the RNAi machinery were not different from that in GC (e.g., Dicer, Argonaute, Piwi; *P*>0.05). The expression of 228 microRNAs, of the 232 analysed, were also unaffected during 4 and 8 d spaceflight (*P*>0.05). In spaceflight, RNAi against green fluorescent protein (*gfp*) reduced chromosomal *gfp* expression in gonad tissue, which was not different from GC. RNAi against *rbx-1* also induced abnormal chromosome segregation in the gonad during spaceflight as on Earth. Finally, culture in RNAi against lysosomal cathepsins prevented degradation of the muscle-specific α-actin protein in both spaceflight and GC conditions.

**Conclusions:**

Treatment with RNAi works as effectively in the space environment as on Earth within multiple tissues, suggesting RNAi may provide an effective tool for combating spaceflight-induced pathologies aboard future long-duration space missions. Furthermore, this is the first demonstration that RNAi can be utilised to block muscle protein degradation, both on Earth and in space.

## Introduction

The RNA interference (RNAi) machinery regulates posttranscriptional gene expression by using small (∼20 nucleotide long) non-coding double-stranded RNA (dsRNA) molecules, in combination with nuclease-containing argonaute complexes, to sequence-specifically silence target mRNAs. Since its discovery [1] RNAi technology has emerged as a promising tool for combating a variety of pathologies. Despite technical difficulties surrounding the effective delivery of dsRNA to diseased tissues, rapid progress has been made towards utilising this technique as a therapeutic over recent years. Indeed, more than a dozen clinical trials are currently underway employing RNAi to target illnesses ranging from cancer to asthma [2], [3].

One currently unexplored use for RNAi technology is within the space (microgravity) environment. A primary aim of the world's space agencies is to send humans to other planetary bodies such as Mars. However, preventing attainment of this goal is the frequent occurrence of various (patho)physiologic adaptations during spaceflight, which may be detrimental for crew health and mission performance. For example, decreases in skeletal muscle mass occur during spaceflight [4]–[6] to levels which are sufficient to impair contractile function [4] and rehabilitation [7]. Loss of bone mass also occurs with 92% of crewmembers experiencing decreases of 5 to 10% [4], [8]. Furthermore, indirect measures of metabolism indicate an increased reliance on glucose utilisation and decreased fat oxidation as a fuel source (reviewed in [9], [10]), which may negatively affect physical performance during extra-vehicular activities requiring prolonged energy expenditure. Finally, T lymphocytes harvested and cultured from crew members display a lowered ability to activate in response to mitogens [11] and also increases in chromosomal aberrations [12], suggesting impaired immune function which, if persistent over longer flights, may pose a serious health risk. Therefore, in order to minimise the inherent risks associated with embarking on long-term deep space explorations, effective countermeasures to these (mal)adaptations must be developed.

Drug-based therapies are limited in spaceflight by their short shelf life due to radiation but RNAi may circumvent this disadvantage due to simple and cheap production of dsRNA in flight. However, a number of factors unique to the space environment render the efficacy of RNAi during spaceflight in question. First, total muscle RNA content is lowered after just 7 days spaceflight in LEO [13]. Thus, because the biogenesis of small RNAs is an integral level of RNAi regulation [14] and since the potency of RNAi is amplified by the replication of cellular RNA [15] it is possible that the RNAi machinery may malfunction in space. Second, RNA is less-stable than DNA and may, therefore, be more susceptible to damage by increased radiation in space which again may reduce the effectiveness of RNAi. Third, RNAi evolved as an immune surveillance system whereby malicious genetic material in the form of viruses and transposons are rapidly targeted for destruction [16]. Since T lymphocytes demonstrate impaired activation and increased chromosome aberrations in space [11], [12], and the transcription of genes integral to effective T-cell immune responses is suppressed [17], it may be logical to hypothesise that the RNAi immune response may also be specifically altered by spaceflight, perhaps in the form of reduced mRNA transcription for components of the RNAi machinery. Indeed, UV radiation significantly affects the mRNA expression of major components of RNAi silencing processes in plants [18]. Therefore, before the space agencies can begin to commit the large sums of money necessary to investigate therapeutic strategies during spaceflight, an important initial step is to demonstrate the efficacy of RNAi in this unique environment. Since the nematode *Caenorhabditis elegans* (*C. elegans*) was the first animal in which RNAi was demonstrated [1] and because we have established *C. elegans* as an *in vivo* model organism for understanding the effects of spaceflight [19]–[22] we tested the efficacy of RNAi in *C. elegans* during spaceflight.

The aim of this experiment was, therefore, to extend the use of *C. elegans* to determine whether the RNAi machinery continues to function normally during spaceflight. We report for the first time the stable expression of microRNAs and mRNA of genes encoding for components of the RNAi machinery during spaceflight. We also show that RNAi treatment in space induces alterations in target protein expression and localisation that are not different from ground controls. Furthermore, treatment with RNAi against lysosomal enzymes during spaceflight prevents the degradation of muscle proteins on return to Earth. Thus, RNAi presents a viable option for targeted therapeutic strategies onboard future long-term manned space explorations.

## Results

### Expression of RNAi machinery and microRNAs are normal after spaceflight

We first examined the expression of mRNAs encoding for components of the RNAi machinery after spaceflight to determine whether spaceflight *per se* may affect cellular capacity to silence genes in response to exogenous dsRNAs. To achieve this we flew L1 larvae in nutrient-deprived media, which were subsequently introduced to a liquid food source upon arrival in microgravity to activate the experiment. Animals were cultured for 8 d before experiment cessation by freezing at −80°C and remained frozen on return to Earth. Identical conditions were conducted on Earth simultaneously (ground control) using the orbital environmental simulator (OES) which adjusts temperature, O_2_ and CO_2_ concentrations, and relative humidity, but not microgravity or radiation levels, to simultaneously match conditions on the International Space Station. Only adult animals (2^nd^ generation) were collected, by sedimentation, and their total RNA was isolated. Microarray analysis revealed normal expression of genes encoding for key proteins involved in the RNAi process after 8 d spaceflight versus ground controls (table 1).

**Table 1:** Gene expression of the RNAi apparatus is unaltered by spaceflight.

RNAi component protein	Gene name	Description	Ground control mRNA expression (av. fold change)	Spaceflight mRNA expression (av. fold change)	Significance
Dicer (RNase III)	*rnh-1.0*	Predicted RNase H	1.01±0.11	1.00±0.10	*P*>0.05
	*rnh-1.1*	RNase H family member	0.47±0.02	0.49±0.06	*P*>0.05
	*rnh-1.2*	RNase H family member	1.29±0.11	1.09±0.33	*P*>0.05
	*rnh-1.3*	RNase H family member	1.03±0.01	1.07±0.09	*P*>0.05
	*rnh-2*	RNase H2 subunit	0.96±0.11	1.07±0.10	*P*>0.05
	*dcr-1*	Dicer family member	0.99±0.04	1.18±0.10	*P*>0.05
	*drh-1*	DExH-box helicase	1.02±0.10	1.12±0.11	*P*>0.05
	*drh-3*	Dicer related helicase family member	0.98±0.07	1.12±0.09	*P*>0.05
PIWI	*ppw-1*	PIWI-domain containing family member	1.11±0.05	1.00±0.06	*P*>0.05
	*ppw-2*	PIWI-domain containing family member	1.01±0.19	0.94±0.11	*P*>0.05
	*prg-1*	PIWI protein	1.07±0.07	1.03±0.05	*P*>0.05
	*rde-1*	PIWI family member	1.07±0.06	1.08±0.09	*P*>0.05
Argonaute	*ergo-1*	Endogenous argonaute family member	1.05±0.07	1.20±0.04	*P*>0.05
	*sago-1*	Argonaute mutant family member	1.03±0.09	0.89±0.05	*P*>0.05
	*sago-2*	Argonaute homolog	1.13±0.13	0.89±0.12	*P*>0.05
RDE-4	*rde-4*	dsRNA binding protein	1.07±0.10	1.19±0.09	*P*>0.05

Adult hermaphrodites (2^nd^ generation) collected at 8 d during spaceflight showed no change in gene expression for components of the RNAi machinery, which were not different ground controls (*P*>0.05). mRNA expression values are the average of 18 separate probes over six microarrays, and are relative to an internal control (1G controls).

We also aimed to determine whether the expression of microRNAs was altered in adult animals after 4 d (1^st^ generation) and 8 d (2^nd^ generation) spaceflight in order to test the capacity of cells to silence-genes in response to aberrant genome-encoded RNA during spaceflight. Of the 232 microRNAs analysed, 228 remained unchanged during spaceflight. Only 4 targets showed a significant change (increase or decrease ±20% in flight versus controls (ground control and 1G centrifuge) in expression after spaceflight: miR-60 +1.33-fold (*P* = 0.038); miR-1819 −0.72-fold (*P* = 0.049); miR-1823 −0.68-fold (*P* = 0.005); miR-2215 −0.76-fold (*P* = 0.003). Thus, congruent with the lack of change in mRNA expression levels, the expression of the vast majority of microRNAs is not affected by microgravity.

### RNAi effectively silences transgenic and endogenous genes in the gonad after 4 d spaceflight

**Figure 1:**
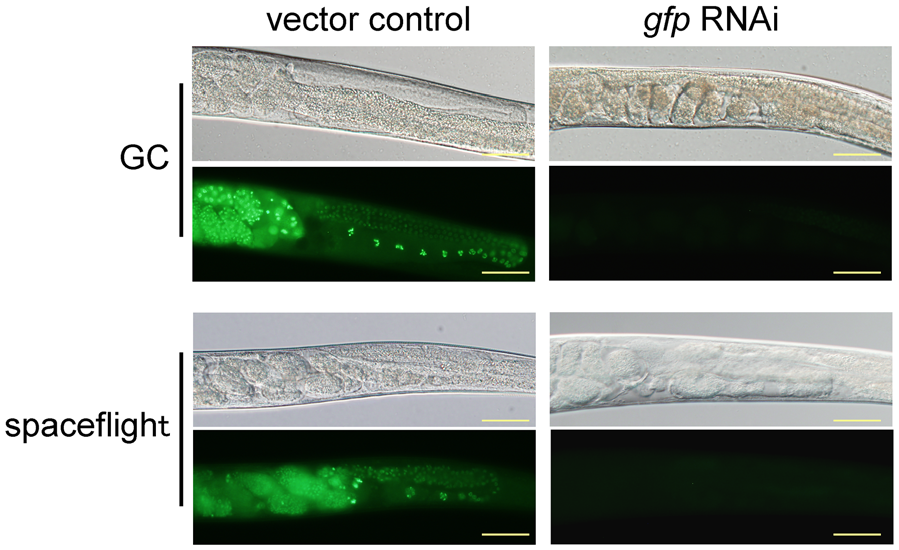
RNAi against *gfp* reduces chromosomal GFP expression in spaceflight and ground control (GC). Animals fed RNAi vector control for 4 d from L1 larvae developed into normal adults in GC and spaceflight conditions. These animals also displayed GFP expression in oocytes and embryos in GC and spaceflight. Animals fed *gfp* RNAi for 4 d also developed normally to adulthood in GC and spaceflight, and demonstrated a loss of GFP expression in both GC and spaceflight. Scale bars represent 50 µm.

**Figure 2:**
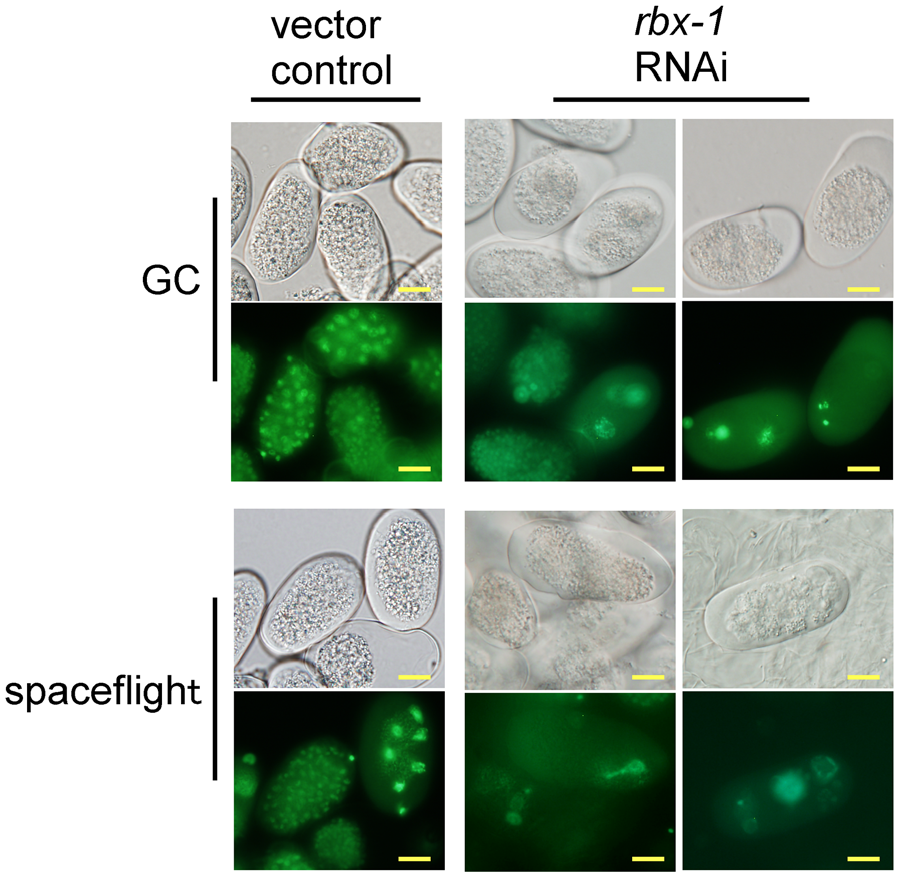
*rbx-1* RNAi induces abnormal chromosomal GFP localisation in spaceflight and ground control (GC). Adult animals fed RNAi vector control from L1 larvae for 4 d produced normal eggs in GC and spaceflight, and display normal embryonic chromosomal GFP localisation in GC and spaceflight. RNAi against *rbx-1* for 4 d caused abnormal embryo development in GC and spaceflight, and induced irregular embryonic nuclear segregation and arrest of meiotic division in both GC and spaceflight. Scale bars represent 10 µm.

To directly determine if RNAi functions normally in spaceflight, L1 larvae of the strain AZ212 (integrated array pAZ132; *pie-1::GFP::histone H2B* fusion, which express histone-tagged green fluorescent protein (GFP) in the nuclei of oocytes and embryos) were prepared as above. Upon arrival in space larvae were grown to adulthood by culturing under three conditions: *gfp* RNAi; *rbx-1* RNAi, and; vector control for 4 d before freezing at −80°C. RNAi against *gfp* was chosen due to its use in the seminal demonstration of the efficacy of RNAi in *C. elegans* [1]. RNAi against *rbx-1* was employed for its previous validation by the authors [23]. Fluorescent light microscopy on return to Earth demonstrated that in vector controls, GFP expression levels were comparable between 4 d spaceflight and ground controls (figure 1). RNAi against *gfp* resulted in decreased embryonic GFP expression that was not different between spaceflight and ground controls (figure 1). Furthermore, in both spaceflight and ground control conditions, RNAi against *rbx-1* induced abnormal embryonic nuclear segregation and arrest of meiotic division observed by histone::GFP localisation (figure 2).

### RNAi against lysosomal protease genes prevents muscle protein degradation after 4 d spaceflight

**Figure 3:**
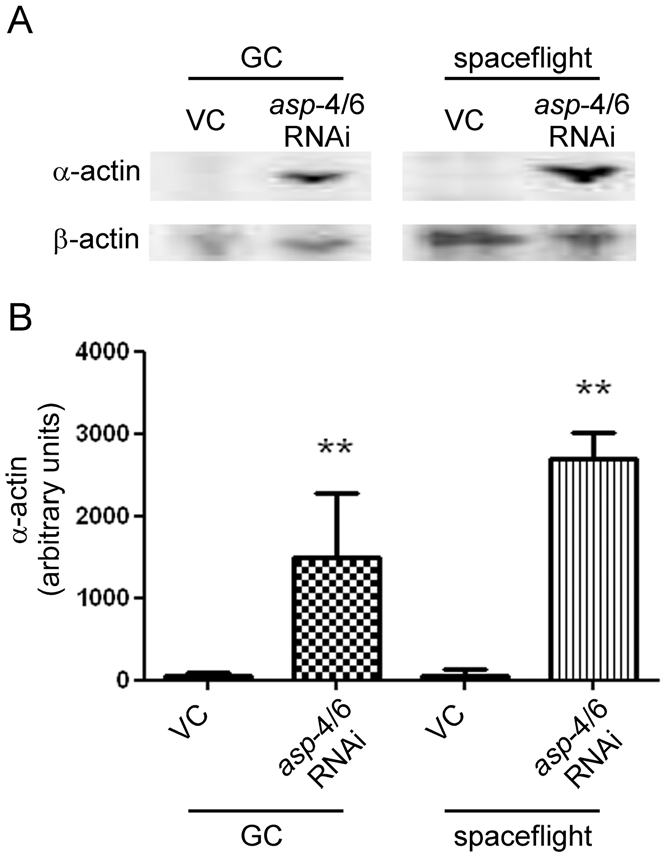
Degradation of α-actin is prevented by *asp-4* and *asp-6* RNAi in spaceflight and ground control (GC). Dauer animals treated for 4 d with RNAi vector control (VC) developed to adulthood. In both GC and spaceflight conditions animals displayed major loss of muscle specific α-actin following lysis in the absence of lysosomal protease inhibitors. Treatment with *asp-4* and *asp-6* RNAi for 4 d in GC and spaceflight resulted in a preservation of α-actin levels. A, representative immunoblot; B, average non-normalised quantification of three Western blots against α-actin. ** denotes significant difference from both GC and spaceflight VC conditions (*P*<0.01).

Finally, to test whether RNAi against lysosomal cathepsins in space (*asp-4*, *asp-6*) prevented the degradation of muscle protein α-actin on return to Earth, dauer animals were flown in liquid media as above. On arrival in space dauers were cultured in either a vector control or *asp-4* and *asp-6* [24] RNAi for 4 d until adulthood. Samples were prepared for Western blot analysis in the presence of a protease inhibitor cocktail, which inhibits the activity of the proteasome, calpains and caspases but not that of lysosomal enzymes. Immunoblotting was performed against α-actin for its specificity to muscle, with β-actin used as a ubiquitously expressed loading control. Multiple Western blots (each blot using a different primary antibody against α-actin) revealed a preservation of α-actin protein levels in animals cultured in the presence of *asp-4* and *asp-6* RNAi versus vector control, in both spaceflight and ground control conditions. A near complete loss of α-actin, within limits of detection, was observed in animals cultured in vector control (figure 3); these observations were found to be statistically significant (P<0.01).

## Discussion

The incidence of spaceflight-induced (patho)physiological adaptations is a major obstacle preventing long-term space exploration. RNAi may provide an effective strategy for therapy, though validation of this technique in the unique space environment is initially required. Here we report for the first time that the RNAi machinery functions as effectively during 4 and 8 d spaceflight as it does on Earth, as evidenced by normal expression of genes encoding for the RNAi machinery and of microRNAs, comparable patterns of GFP transgene abnormalities with RNAi treatment, and inhibition of α-actin protein degradation with lysosome-targeting RNAi in both spaceflight and ground control conditions.

The finding that expression of genes involved in the RNAi apparatus (e.g. Dicer, Argonaute and PIWI) is normal during spaceflight indicates that the RNAi machinery maintains the capacity to function normally. Thus, earlier reports of reduced global RNA synthesis after 7 d spaceflight [13] do not appear to be reflected at the level of mRNA transcription, at least of genes coding for RNAi components. Additionally, we report here that the expression of 228 of the 232 microRNAs analysed is unaffected in spaceflight samples. It appears therefore that factors unique to spaceflight, for example microgravity and increased radiation exposure do not impair cellular ability to recognise and initiate gene silencing in response to both exogenous and endogenous genetic material.

We also demonstrate that treatment with *gfp* RNAi in space causes reduced gonad GFP expression to levels comparable to those found in Earth-based controls. GFP expression was, however, normal in animals fed a vector control, therein excluding the possibility that lowered protein synthetic capacity in space [19] may have influenced GFP levels under RNAi conditions. Thus, assuming identical rates of protein degradation in RNAi and vector control conditions, the observed reduction of GFP levels can be attributed to actions of the RNAi machinery. Furthermore, *rbx-1* RNAi induced abnormal nuclear histone::GFP localisation within the gonad in both spaceflight and ground controls. Combined, these results indicate that not only is expression of genes involved in RNAi normal but that the protein products of these genes continue to function normally in spaceflight. Thus, spaceflight does not induce negative regulatory post-transcriptional or post-translational modifications that impair the efficiency of RNAi.

Finally, the efficacy of RNAi in space is illustrated by the inhibition of muscle-specific protein (α-actin) degradation by *asp-4* and *asp-6* RNAi treatment, despite virtual total loss of α-actin levels in vector control animals. Thus, cathepsin RNAi administered during spaceflight effectively silences lysosomal proteolytic activity. Furthermore, it is apparent that spaceflight *per se* does not have large negative effects on lysosomal proteases since degradation of α-actin occurred in the vector control condition. This is also, to our knowledge, the first report that RNAi can be used to block the degradation of muscle proteins on Earth and in space. Because loss of muscle mass implies an increase in the degradation of pre-existing proteins, this result indicates that RNAi may be developed to combat conditions of muscle wasting both on Earth and in space.

Taken together, these results demonstrate that, despite reports of impaired T cell function during spaceflight aboard the International Space Station [11], [12], the RNAi immune response is unaffected at the level of both mRNA expression and protein function. Demonstration that this fundamental immunological process is preserved is especially important within the space environment. Enhanced pathogenic virulence in space flown bacteria and increased microbe growth rates [25] render it vital that cells retain their ability to cope with increased exposure to virulent microbes, and indicates that crew member health in response to such stressors may be sustained on long-term exploratory missions.

These experiments also show that RNAi works effectively in at least two tissues; muscle and the gonad. Muscle is a key site for many of the potentially negative consequences of spaceflight to crew member health; for example muscle wasting and altered metabolism towards increased glucose utilisation. The gonad is a primary site for the effects of increased radiation-induced DNA damage and subsequent heritability of genetic defects. Thus, application of RNAi in these tissues may represent a viable therapeutic possibility for future missions to overcome these problems. It is also likely, in light of the lack of change in global mRNA expression of the RNAi apparatus, that RNAi may be as effective in other tissues, therein promoting the use of RNAi to combat a large range of pathologies in space.

In conclusion, we report for the first time the efficacy of RNAi during spaceflight within multiple tissues. However, whether this is true outside of Low Earth Orbit where the effects of radiation are increased is currently unknown, though *C. elegans* provides a practical and cost-effective model organism for incorporation on future unmanned deep space explorations in which to answer this, and other, questions of biological importance. Nevertheless, RNAi presents a potential therapeutic strategy for combating deleterious spaceflight-induced adaptations, for maintained crew health and mission performance. Indeed, we show that protein degradation; a key element underlying muscle wasting, can be prevented by the application of RNAi against proteolytic enzymes on Earth and in space. As such, achieving long-duration explorations of deep space may be made more feasible through the use of RNAi technology to overcome the numerous threats posed to human health by prolonged exposure to the space environment.

## Materials and Methods

### Nematode and bacteria preparation

Two developmental stages of *C. elegans* were cultured for these experiments; L1 and dauer larvae. To provide L1 larvae nematode eggs were prepared using the alkaline bleach method with 0.5 N KOH and 1.0% NaClO. After overnight incubation in M9 buffer containing 5 mg/L cholesterol at 20°C, the hatched L1 larvae were used for the space experiment. L1 larvae were prepared from the strain AZ212 (ruIs32; *unc-119 (ed3)*; [26]), whose integrated array is pAZ132 (*pie-1::GFP::histone H2B* fusion and *unc-119* subclone). AZ212 GFP signals are revealed in the nuclei of oocytes and eggs. To provide dauer larvae animals were cultured according to the protocol [27] with the exception that animals were cultured on 8× peptone NGM agar plates. Dauer animals were prepared from the strain PD55 (*tra-3*(e1107)IV; ccIs55V) whose integrated transgene ccIs55 consists of 58- portions of wild-type *unc-54* (muscle myosin heavy-chain) gene, fused in to the *lacZ* gene of *E. coli*, followed by a 38-terminal portion of the *unc-54* gene. The *unc-54*::*lacZ* fusion encodes a 146-kDa polypeptide.

Double stranded RNA of *gfp* (*gfp* fragment of pGFP U17997 (Clonthech) digested with *Hind*III and *Eco*RI), *rbx-1* [23], *asp-4* [24] and *asp-6* (Ahringer library, clone V-5N20) genes were synthesized in *Escherichia coli* HT115 (DE3) with Litmus 28 plasmid vector *in vivo* system [23]. The vector control used was *Escherichia coli* HT115 (DE3). *E. coli* bacterial feeds were cultured in S basal medium to an OD600 of approximately 3.5.

### Experimental design

A full description of the experimental procedures and flight hardware, including illustrations, employed on this experiment can be found in [28]. We tested three experimental conditions in each culture: ground control, 1G (control) centrifuge onboard the International Space Station (ISS) and micro-gravity samples onboard ISS. Briefly, for the microarray gene expression and microRNA expression experiments N2 wild type L1 larvae were used. For the *gfp* and *rbx-1* RNAi experiments AZ212 L1 larvae were used. For the *asp-4* and *asp-6* RNAi experiment PD55 dauer larvae were used. All larvae were maintained in stasis within S basal medium until initiation of the experiment. The experiment was activated once onboard the ISS by reintroducing animals to food. All experiments were carried out under temperature controlled conditions in the Cell Biology Experiment Facility (CBEF) within the KIBO module. Cultures for microarray analysis were grown for 8 d. Cultures for microRNA expression analysis were grown for 4 d and 8 d. Cultures for RNAi treatment experiments were grown for 4 d. Experiments were stopped by first observing the animals by light microscopy and then by freezing with subsequent storage at −80°C in MELFI. Microscopy at the end of culturing confirmed that the 4 day cultures grew to adulthood as the first generation for both the L1 and dauer cultures, and that the 8 day cultures grew to a second generation of adulthood.

### Microarray and microRNA assay

Flight samples were thawed on ice with M9 buffer containing 0.05% gelatine, and approximately 10^3^ adult hermaphrodites were collected in each experimental condition by picking under stereo microscopy or by sedimentation on ice. Total RNA was isolated with TRIzol Reagent (Invitrogen). Global gene expression and entire microRNA expression were analyzed using the Agilent *Caenorhabditis elegans* Oligo Microarray 22K (Agilent) and Filgen® Array miRNA *Caenorhabditis elegans* 232 probes (Filgen), respectively. Biological triplicates were used for the global gene expression array experiments, and these were each analysed over six microarrays. All global gene expression and microRNA data are MAIME compliant and deposited in the gene expression omnibus (GEO) database (accession numbers GSE27338 and GSE27288 for global gene expression and microRNA data, respectively). In the microRNA expression analysis, we compared the expression data between two groups: micro-gravity samples (4 d and 8 d) and controls (4 d and 8 d ground control and 1 G centrifuge onboard ISS). All statistical analyses were carried out by one-way ANOVA with significance set at *P*<0.05.

### Fluorescent light microscopy

Flight samples were thawed on ice with M9 buffer containing 0.05% gelatine, and developed adult hermaphrodites plus eggs laid were picked onto HTC Super Cured ® slides (Thermo Scientific). GFP visualization was performed under constant excitation light and the same exposure period for fluorescence with a fluorescent microscope (BX51; Olympus) and a CCD camera (DP70; Olympus).

### Immunoblotting

Upon return to Earth, developed dauer animals were thawed on ice in the presence of a protease inhibitor cocktail (Complete Mini protease inhibitor tablet; Roche Diagnostics, Mannheim, Germany) in 15 ml tubes. Samples were then pelleted by centrifugation at 13,000×g for 10 min at 4°C and the supernatant removed to leave ∼1 ml. Samples were then transferred to 1.5 ml tubes and spun at 13,000×g for 3 min. The supernatant was then removed to leave 400 µl, the pellet homogenised and 200 µl 3×Laemmli buffer added. Samples were then boiled for 5 min and stored at −20°C until analysis. Samples were thawed on ice, vortexed thoroughly and centrifuged at 13,000×g for 3 min at 4°C to pellet any residual *E. coli* bacteria. Samples (5 µl) were loaded onto a precast 26-well 12% sodium dodecyl sulfate polyacrylamide electrophoresis gel (Criterion XT Bis-Tris; Bio-Rad, Hemel Hempstead, UK) and ran at 200 V for 1 h. After equilibration in transfer buffer for 15 min, the gel was transferred on ice at 100 V for 45 min to a methanol pre-wetted 0.2 µm Immobilon PVDF membrane (Millipore, Billerica, MA). Next, the membrane was blocked in 5% (w/v) BSA in TBS-T (Tris Buffered Saline and 0.1% Tween-20) for 1 h at room temperature and then incubated overnight at 4°C in primary β-actin antibody (New England Biolabs, UK) at a 1∶3000 dilution in 5% (w/v) BSA in TBS-T. The following morning the membrane was washed (3×5 min) in TBS-T and then incubated in anti-rabbit secondary antibody (New England Biolabs, UK) at a 1∶2000 dilution in 5% BSA / TBS-T for 1 h at room temperature. After further washes (3×5 min) in TBS-T the membrane was developed using Immunstar ECL reagent (Bio-Rad, Richmond, CA) for 5 min and the protein bands visualised on a Chemidoc XRS system (Bio-Rad, Hercules, CA). The membrane was subsequently washed (rapidly×2, then 3×5 min) in TBS-T before stripping antibody-bound proteins in neat reagent (Thermo Scientific, Rockford, IL) for 15 min. The membrane was then re-probed with primary α-actin antibody (Sigma-Aldrich, St. Louis, MO) at a 1∶3000 dilution in 5% (w/v) BSA in TBS-T overnight at 4°C. The next day washes, secondary incubation and visualisation were performed as above. This method was repeated another two times with different primary antibodies against α-actin (Invitrogen, Paisley, UK; DSHB, Iowa, US) for a total of three Western blots. Peak density from the three runs was statistically analysed by two-way repeated measures ANOVA with the level of significance set at *P*<0.01.
